# Breastfeeding, socioeconomic status, and long-term postpartum weight retention

**DOI:** 10.1186/s13006-022-00534-0

**Published:** 2023-01-05

**Authors:** Saralyn F. Foster, Christian Vazquez, Catherine Cubbin, Amy R. Nichols, Rachel R. Rickman, Elizabeth M. Widen

**Affiliations:** 1 Department of Nutritional Sciences, The University of Texas at Austin, 200 W. 24th Street, A2703, Austin, United States; 2 Steve Hicks School of Social Work, The University of Texas at Austin, 1925 San Jacinto Blvd, Austin, United States; 3 School of Social Work, The University of Texas at Arlington, 211 S Cooper St Arlington, Arlington, United States; 4 Departments of Population Health and Health Social Work, Dell Medical School, The University of Texas at Austin, 1601 Trinity Street, Austin, United States; 5 Departments of Women’s Health & Pediatrics, Dell Pediatric Research Institute, The University of Texas at Austin, 1400 Barbara Jordan Blvd, Austin, United States

**Keywords:** Breastfeeding, Socio-economic status, Postpartum weight, BMI, Maternal health

## Abstract

**Background:**

Almost half of all pregnant women in the United States gain weight above Institute of Medicine gestational weight gain guidelines. Breastfeeding has been shown to reduce weight retention in the first year postpartum; however, women with lower socioeconomic status (SES) tend to initiate breastfeeding less often than women with higher SES. We investigated associations between duration of breastfeeding with mother’s long-term postpartum weight status at 4–10 years and evaluated whether the associations varied by SES.

**Methods:**

Maternal and infant dyads (*N* = 2144 dyads) are from the Geographic Research on Wellbeing survey (GROW), 2012–2013, a long-term, cross-sectional follow-up of the Maternal and Infant Health Assessment (MIHA) based in California, USA. Pre-pregnancy body mass index (BMI) was obtained from self-report of height and weight during MIHA, while breastfeeding history and self-report of current body weight was collected at the 4–10 year GROW postpartum visit. SES score was derived from a composite score of percent federal poverty level and education and was dichotomized into High and Low SES groups at a score of three. Multivariable linear regression was used to examine association between breastfeeding and maternal weight status, and to examine for effect modification by SES.

**Results:**

Average long-term weight retention 4–10 years postpartum was 4.0 kg. Fewer lower SES vs. higher SES women breast fed at least six months (51% versus 70%, *p* < .001) or ever breastfed (74% versus 89%, *P* < .001). Women who breastfed at least six months had lower long-term postpartum weight retention compared to those who did not (*b* = -1.06 kg, (-1.93, 0.25); *p* = 0.01); however, these association did not vary by SES.

**Conclusion:**

Six months of breastfeeding is associated with lower BMI at 4–10 years and lower body weight, and effects do not vary by SES. Future policies and guidelines should consider building an infrastructure that is supportive of longer breastfeeding duration. Moreover, further research is needed to identify the impact of additional behavioral and environmental factors on long-term maternal weight status. Understanding the drivers of excessive weight retention pospartum can help us not only improve the pregnant person’s health but the health of their children.

## Background

Almost half of all women in the United States have excessive pregnancy weight gain [1], which is associated with adverse short- and long-term health outcomes for mothers and their children, including greater postpartum weight retention [2, 3]. Postpartum weight retention, the weight change from pre-pregnancy to a period postpartum (typically > 6 months), is associated with a higher risk of overweight and obesity, cardiovascular disease, and diabetes [4]. Studies have shown that excessive postpartum weight retention is common one to two years postpartum [5, 6].

Breastfeeding is one potentially modifiable factor that can lead to less postpartum weight retention in the early postpartum period [7]. The World Health Organization (WHO) recommends 6 months of exclusive breastfeeding with continued breastfeeding up to two years of life [8]. Breastfeeding confers many benefits to not only the child but the mother as well [9, 10], such as reduced risk of type 2 diabetes, certain breast cancers, and ovarian cancer [11, 12]. Recent research has examined breastfeeding’s association with postpartum weight retention with breastfeeding characterized in various ways including duration (months or weeks) or ever breastfeeding [13, 14]. However, it is unknown whether effects of breastfeeding duration on weight retention and long-term body size vary by socioeconomic status (SES).

In the United States, women with lower SES are less likely to initiate and have shorter durations of breastfeeding [15, 16, 17], and, similarly, lower education levels are associated with lower breastfeeding initiation [17]. These women may also have less access to physical activity/green spaces and have less access to higher quality diets [18, 19]. Together this may lead to a higher risk for long-term weight retention and excess adiposity.

We sought to examine associations between previous breastfeeding with long-term postpartum weight retention and body mass index (BMI) in women who participated in the Geographic Research on Wellbeing (GROW) study, and further, to evaluate whether associations varied by SES. We hypothesized that longer duration of breastfeeding would be associated with lower long-term weight retention and BMI, and additionally that effects of breastfeeding on long-term weight status would vary by SES with those in the higher SES group less weight retention than those in the lower SES group.

## Methods

### Study design and subjects

 Data were used from the Geographic Research on Wellbeing (GROW) survey of 3,016 women, which has been previously described in detail [20]. GROW was a cross-sectional follow-up study of the annual, cross-sectional, statewide-representative Maternal and Infant Health Assessment (MIHA) in California, United States of America. GROW was conducted from 2012–2013 and included follow-up MIHA participants (2003–2007) that were 4 to 10 years postpartum. For the GROW survey, participants were directed to answer questions about the index pregnancy/child surveyed in MIHA. Participants were eligible for MIHA, the state of California’s version of the CDC’s Pregnancy Risk Assessment Monitoring System, if they were 15 years or older, English or Spanish speaking (the available languages for the survey), and California residents, per guidelines set by the state. Participants were selected according to the following strata: region of residence, education, and race/ethnicity, with oversampling for African Americans, to ensure appropriate generalizability to the state of Califorina, to obtain adequate representation of an understudied group as well as to examine health disparities and have accurate estimates. MIHA response rates exceeded 70% each year. Participants for GROW were chosen from the six urbanized counties with the highest number of MIHA respondents across California: Alameda, Los Angeles, Orange, Sacramento, San Diego, and Santa Clara. During MIHA (2003–2007), pre-pregnancy BMI, total gestational weight gain (GWG), maternal age at infant’s birth, gestational age, weight, height, education, and income were obtained from self-report. As part of GROW, questionnaires requesting breastfeeding history, additional pregnancies, perception of self-reported health (excellent, good, fair, poor) and self-report of current body weight were completed. To reduce selection bias in sampling for GROW, participants were chosen randomly with stratification by education and language. The GROW sample has previously been shown to be highly representative to the target population of all women in California giving birth during the study time period [20].

**Fig. 1:**
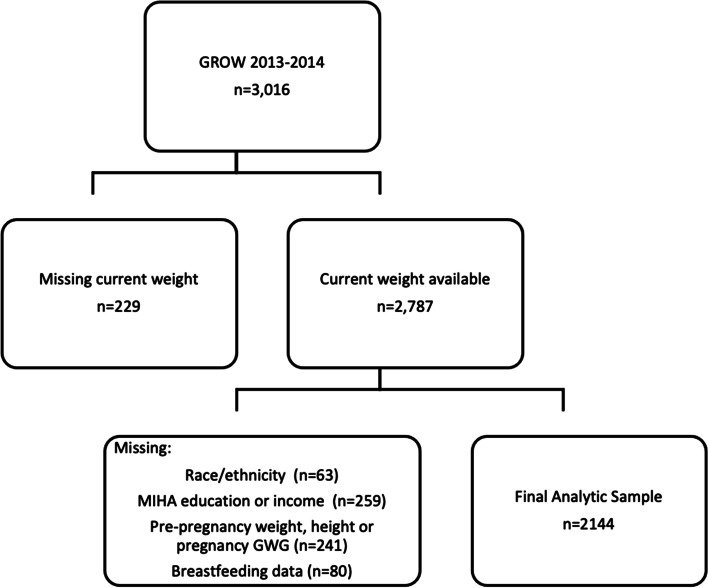
Participant flow diagram

This complete-case analysis focuses on participants who were followed in MIHA who had information on pre-pregnancy body size (weight and height), covariates and who had complete data on breastfeeding and postpartum body size assessed via GROW (Fig. 1).

### Breastfeeding

The primary predictor, breastfeeding, was categorized in three ways: 1) ever breastfed [21], 2) breastfed at least six months [22], 3) duration in months [23]. Ever breastfed and breastfeeding at six months were dichotomous variables (yes/no). Ever breastfed was “yes” if the infant was breastfed a minimum of one month. Breastfeeding at six months was “yes” if the mother breastfed at least six months.

### Socioeconomic status

Self-reported education and family income were used to derive the composite socioeconomic status (SES) score, as has been done previously with GROW data [20]. Briefly, education and income were each given a score of 1–4 (did not complete high school, high school graduate/GED, some college, college graduate and less than 100% of the federal poverty level [FPL], 101–200% FPL, 201–300% FPL, and 301 + % FPL) with higher scores indicating higher values. The two scores were then added together and divided by two. A score of 3 or higher indicated High SES while a score below 3 indicated Low SES.

#### Weight outcomes

Postpartum BMI was calculated from self-reported weight and height, while weight retention was calculated by subtracting self-reported weight from GROW (2012–2013) from the self-reported pre-pregnancy weight from MIHA. Gestational weight gain (GWG) z-scores, standardized measurements of maternal pregnancy weight gain independent of duration of gestation, were calculated per previous published methods [24].

### Statistical analyses

Analyses were conducted using SAS version 9.4 (SAS Institute Inc; Cary, NC), and accounted for the complex sample design. An alpha of 0.05 was used for statistical tests with 95% confidence intervals.

A series of multivariable linear regression models were fitted to examine how breastfeeding exposures related to long-term BMI and postpartum weight retention among all participants, and then also stratified by SES. A set of covariates were examined for potential confounding by examining whether inclusion of the covariate changed the effect sizes of breastfeeding exposures by > 10% [25]. Our base model included adjustment for covariates: maternal age at delivery, perception of self-related health, time postpartum at the GROW visit, race/ethnicity, and parity. To examine for sensitivity of our findings to prenatal exposures, we also fit models with additional adjustment for prenatal factors, including pre-pregnancy BMI and GWG z-scores.

The Institutional Review Boards at The University of Texas at Austin, the University of California, San Francisco, and the California Department of Public Health approved study procedures for GROW. All participants gave informed consent.

## Results

A total of 2144 women were included in the analysis (Fig. 1). Demographic data can be found in Table1. Women in the Low SES group on average were younger compared to High SES women, and more often from a minoritized population compared to white women. The average duration of breastfeeding in the total sample was eight months, with Low SES mothers breastfeeding two months shorter compared to High SES mothers. Accordingly, fewer Low vs. High SES women ever breastfeed or breastfed at six months.

**Table 1:** Participant characteristics, GROW 2012–2013

	All *N* = 2144	Low SES *N* = 1048	High SES *N* = 1096	*p*-value
Mean age at birth, years	29.0 ± 0.15	26.6 ± 0.20	32.5 ± 0.16	< .0001
Mean Gestational age at birth, weeks	38.0 ± 0.05	38.0 ± 0.07	38.0 ± 0.07	0.77
Ever breastfed, N (%)	1751 (81)	773 (74)	978 (89)	< .0001
Months breastfeeding, average	8.0 ± 0.20	7.5 ± 0.28	9.8 ± 0.3	< .0001
Breastfed at least 6 months, N (%)	1306 (61)	533 (51)	773 (70)	< .0001
Excellent/good self-rated health, N (%)	1772 (83)	769 (73)	1003 (91)	< .0001
Race, N (%)				< .0001
Asian	248 (12)	56 (5)	192 (17)	
Black	268 (12)	171(16)	97 (9)	
Latina – Foreign born	434 (20)	403 (39)	31 (3)	
Latina – US born	360 (17)	238 (23)	122 (11)	
White	834 (39)	180 (17)	654 (60)	
Education Level, N (%)				
Less than High School	365 (17)	365 (35)	0	
High School/GED	410 (19)	368 (35)	42 (4)	
Some College	392 (18)	242 (23)	150 (14)	
College Graduate	977 (46)	73 (7)	904 (82)	
Percent federal poverty level, N(%)				
0–100	481 (22)	481 (46)	0	
101–200	402 (19)	402 (38)	0	
201–300	213 (10)	123 (12)	90 (8)	
301–400	1048 (49)	42 (4)	1006 (92)	
Parity, N (%)				< .0001
1	847 (39)	348 (33)	499 (46)	
2 to 4	1213 (57)	628 (60)	585 (53)	
5 or more	84 (4)	72 (7)	12 (1)	

Sixty-one percent of the participants started pre-pregnancy with a normal or underweight BMI; with 24% in the overweight category and 15% with obesity (Table 2). When stratified by SES, Low SES participants more often had pre-pregnancy BMI categories of overweight and obesity compared to those in the High SES group (overweight 27% vs 21%; obesity 22% vs 9%). Average pre-pregnancy BMI was higher and in the overweight category for the Low SES group and was lower and in the normal weight category for the High SES group (*p* < 0.0001). Overall, Low SES women started pregnancy 3.7 kg heavier and with higher pre-pregnancy BMI values and had greater long-term weight retention and higher BMI values postpartum.

**Table 2:** Weight characteristics before, during and after the index pregnancy from GROW 2012–2013

	All *N* = 2144	Low SES *N* = 1048	High SES *N* = 1096	*p*-value
Pre-pregnancy BMI, kg/m^2^	24.9 ± 0.13	25.9 ± 0.2	23.5 ± 0.14	< .0001
Pre-pregnancy weight (kg)	65.6 ± 0.37	67.1 ± 0.56	63.4 ± 0.44	< .0001
Total gestational weight gain, kg	14.2 ± 0.15	13.6 ± 0.23	15.0 ± 0.18	< .0001
GWG z-score	-0.23 ± 0.02	-0.3 ± 0.04	-0.13 ± 0.03	0.0005
IOM GWG Recommendations, N(%)				
Below	355 (17)	214 (20)	141 (13)	
Within	813 (38)	359 (34)	454 (41)	
Exceed	976 (45)	475 (45)	501 (46)	
IOM Recommendations by BMI, N(%)				
Pre-pregnancy BMI status: Under/Normal				
Below guidelines	252 (19)	135 (25)	117 (15)	
Within guidelines	565 (44)	202 (38)	363 (47)	
Exceed Guidelines	482 (37)	193 (36)	289 (38)	
Pre-pregnancy BMI status: Overweight				
Below guidelines	44 (8)	37 (13)	7 (3)	
Within guidelines	155 (31)	86 (30)	69 (30)	
Exceed Guidelines	321 (61)	166 (57)	155 (67)	
Pre-pregnancy BMI status: Obesity Class 1				
Below guidelines	30 (15)	22 (16)	8 (13)	
Within guidelines	50 (25)	39 (28)	11 (18)	
Exceed Guidelines	120 (60)	77 (56)	43 (69)	
Pre-pregnancy BMI status: Obesity Class 2/3				
Below guidelines	29 (23)	20 (22)	9 (27)	
Within guidelines	43 (34)	32 (35)	11 (32)	
Exceed Guidelines	53 (42)	39 (43)	14 (41)	
Post-partum weight retention, kg	4.02 ± 0.21	5.68 ± 0.33	1.83 ± 0.21	< 0.001
Long term BMI, kg/m2	26.5 ± 0.14	28.2 ± 0.21	24.3 ± 0.15	< 0.001

Unadjusted regression models examining breastfeeding with these outcomes are shown in Tables 3 and 4. Despite differences in breastfeeding duration by SES, no SES differences between breastfeeding and long-term body size were observed for both long term BMI and long-term weight retention for any of the breastfeeding categorizations after adjustment for covariates (Tables 3 & 4). Overall, ever breastfeeding, and overall duration of breastfeeding were not associated with weight retention or long-term BMI. However, among all women, compared to women who did not breastfeed, 6 months of breastfeeding was associated with smaller long-term body size. Those who breastfed at least six months had an estimated 0.4 units lower BMI and 4 kg less weight retention. Additional model sets were evaluated with additional adjustment for pre-pregnancy BMI and another set of models with adjustment for both prepregnancy BMI and gestational weight gain. The addition of these prenatal and pregnancy related charactertiscs to the model did not meaningfully change the effect size for each of the breastfeeding exposures (Tables 3 & 4: Model Sets 2 & 3).

**Table 3:** Linear regression models showing associations between breastfeeding and long-term weight retention at 4 to 10 years postpartum

	Long-term weight retention at 4–10 years postpartum, kg					
	Unadjusted			Adjusted		
	All β (95% CI)	Low SES β (95% CI)	High SES β (95% CI)	All β (95% CI)	Low SES β (95% CI)	High SES β (95% CI)
Model Set 1: Breastfeeding						
Ever Breastfed	-1.29 (-2.50, -0.08)	-0.84 (-2.36, 0.67)	0.76 (-1.33, 2.86)	-0.85 (-2.04, 0.34)	-0.60 (-2.1, 0.89)	0.13 (-1.67, 1.92)
Breastfeeding Duration, months	-0.06 (-0.12, -0.01)	-0.02 (-0.11, -0.06)	-0.05 (-0.10, 0.01)	-0.03 (-0.09, 0.01)	-0.02 (-0.1, 0.06)	-0.04 (-0.09, 0.01)
Breastfed at least 6 months	-1.58 (-2.47, -0.69)	-0.96 (-2.27, 0.34)	-0.72 (-1.77, 0.33)	-1.09 (-1.93, -0.25)	-0.83 (-2.1, 0.44)	-0.85 (-1.79, 0.09)
Model Set 2: Breastfeeding + Pre-pregnancy BMI						
Ever Breastfed	-1.78 (-2.98, -0.58)	-1.07 (-2.58, 0.44)	-0.01 (-1.89, 1.88)	-0.81 (-2.0, 0.38)	-0.55 (-2.04, 0.94)	0.12 (-1.68, 1.92)
Breastfeeding Duration, months	-0.08 (-0.13, -0.03)	-0.03 (-0.11, 0.04)	-0.06 (-0.11, 0.01)	-0.03 (-0.09, 0.02)	-0.02 (-0.1, 0.06)	-0.04 (-0.09, 0.01)
Breastfed at least 6 months	-1.97 (-2.83, -1.10)	-1.15 (-2.43, 0.12)	-1.17 (-2.15, -0.19)	-1.08 (-1.92, -0.24)	-0.82 (-2.08, 0.45)	-0.85 (-1.79, 0.09)
Model Set 3: Breastfeeding + Pre-pregnancy BMI + GWG (z-score)						
Ever Breastfed	-1.81 (-3.00, -0.63)	-1.00 (-2.47, 0.46)	-0.06 (-1.97, 1.85)	-0.79 (-1.98, 0.4)	-0.54 (-2.04, 0.95)	0.15 (-1.65, 1.96)
Breastfeeding Duration, months	-0.08 (-0.13, -0.03)	-0.04 (-0.12, 0.04)	-0.05 (-0.11, -0.003)	-0.03 (-0.09, 0.02)	-0.01 (-0.09, 0.06)	-0.04 (-0.09, 0.01)
Breastfed at least 6 months	-1.96 (-2.82, -1.11)	-1.2 (-2.45, 0.04)	-1.06 (-2.05, -0.07)	-1.06 (-1.9, -0.22)	-0.80 (-2.1, 0.46)	-0.85 (-1.79, 0.09)

Adjusted model included adjustment for maternal age at delivery, perception of self-related health, time postpartum at the GROW visit, race/ethnicity, and parity. Each Beta-coefficient is showing a separate model

**Table 4:** Linear regression models evaluating associations between between breastfeeding and long term body mass index (BMI) at 4–10 years postpartum, stratified by SES

	Body Mass Index at 4 to 10 years postpartum, kg/m^2^					
	Unadjusted			Adjusted		
	All β (95% CI)	Low SES β (95% CI)	High SES β (95% CI)	All β (95% CI)	Low SES β (95% CI)	High SES β (95% CI)
Model Set 1: Breastfeeding						
Ever Breastfed	-2.05 (-2.87,-1.24)	-0.71 (-1.71, 0.29)	-2.27 (-3.54, -1.01)	-0.33 (-0.79, 0.12)	-0.29 (-0.86, 0.28)	0.03 (-0.65, 0.71)
Breastfeeding Duration, months	-0.07 (-0.11, -0.04)	-0.03 (-0.08, 0.02)	-0.06 (-0.10, -0.02)	-0.01 (-0.03, 0.01)	-0.01 (-0.04, 0.02)	-0.02 (-0.03, 0.002)
Breastfed at least 6 months	-1.92 (-2.51, -1.32)	-0.86 (-1.69, -0.03)	-1.70 (-2.43, -0.98)	-0.41 (-0.74, -0.08)	-0.34 (-0.84, 0.15)	-0.33 (-0.68, 0.03)
Model Set 2: Breastfeeding + Pre-pregnancy BMI						
Ever Breastfed	-0.64 (-1.15, -0.14)	-0.27 (-0.92, 0.38)	-0.01 (-0.72, 0.70)	-0.31 (-0.76, 0.15)	-0.25 (-0.83, 0.32)	0.04 (-0.64, 0.72)
Breastfeeding Duration, months	-0.03 (-0.05, -0.01)	-0.01 (-0.04, 0.02)	-0.02 (-0.04, -0.01)	-0.01 (-0.03, 0.01)	-0.004 (-0.03,0.03)	-0.02 (-0.04,0.002)
Breastfed at least 6 months	-0.84 (-1.21, -0.47)	-0.50 (-1.04, 0.03)	-0.46 (-0.84, -0.9)	-0.4 (-0.72, -0.08)	-0.33 (-0.82, 0.15)	-0.33 (-0.68, 0.03)
Model Set 3: Breastfeeding + Pre-pregnancy BMI + GWG (z-score)						
Ever Breastfed	-0.65 (-1.15,-0.16)	-0.24 (-0.87, 0.38)	-0.03 (-0.74, 0.69)	-0.3 (-0.76, 0.16)	-0.25 (-0.82, 0.32)	0.05 (-0.63, 0.73)
Breastfeeding Duration, months	-0.03 (-0.05,-0.01)	-0.01 (-0.04, 0.02)	-0.02 (-0.04, -0.004)	-0.01 (-0.03, 0.01)	-0.003 (-0.03,0.03)	-0.02 (-0.04,0.002)
Breastfed at least 6 months	-0.84 (-1.20, -0.47)	-0.52 (-1.04, -0.01)	-0.43 (-0.81, -0.05)	-0.40 (-0.72, -0.08)	-0.33 (-0.81, 0.16)	-0.32 (-0.68, 0.03)

Models adjusted for maternal age at delivery, perception of self-related health, time postpartum at the GROW visit, race/ethnicity, and parity

## Discussion

In our study of 2144 women from the diverse, representative Geographic Research on Wellbeing (GROW) study, we examined whether duration of breastfeeding was associated with long-term postpartum weight retention and maternal BMI, and whether these associations differed by SES. Our research is novel for its sample size of a diverse group of women with postpartum follow-up up to 10 years. Average duration of breastfeeding was eight months, and those with lower SES tended to have high pre-pregnancy BMI and retained more weight during the long-term postpartum period. Overall, we found that six months of breastfeeding was associated with lower weight retention and BMI in women at 4–10 years postpartum. Differences by SES were observed in breastfeeding duration, but no differences were observed between any category of breastfeeding and long-term body size or postpartum weight retention.

These finding did not support our primary hypothesis that effects of breastfeeding on postpartum weight retention (kg) or body mass index (kg/m^2^) would be different between the High and Low SES groups. One reason may be that race and ethnicity are greater drivers of weight retention and once accounted for in the model, ameliorate the effect of SES. For example, 2016 LAMP Follow-Up project of 1524 women from the Los Angeles area found that those women of Hispanic ethnicity had 1.5 times the odds of having postpartum weight retention at two years compared to non-Hispanic women after controlling for age, education, and pre-pregnancy BMI [26]. In addition to SES, race, and ethnicity, education and job status have been shown to affect maternal weight and breastfeeding status. Archuleta and Chao [26] found at two years postpartum that women who only had a high school education or lost a job during pregnancy had 1.77 or 2.62 the odds of retaining postpartum weight compared to those with more than a high school education or no stress from job loss.

For our second hypothesis, that breastfeeding would be associated with postpartum weight retention and BMI, we found that six months of breastfeeding was associated with lower weight retention and body mass index in women 4–10 years postpartum. Similar to our results, other studies found that breastfeeding for six months was associated with reduction in postpartum weight retention [7, 27, 28]. The first six months of breastfeeding comprises the most intensive months of breastfeeding because it is prior to the introduction of complementary foods, thus is the sole source of nutrition and encompasses the infant’s highest growth velocity. Mazariegos and colleagues [28] found in their cohort of 75,421 Mexican women had lower weight gain through 6 months of breastfeeding. A 2018 meta-analysis by Jiang et al. found significant differences between post-partum weight retention and breastfeeding for 6 months with those breastfeeding for 6 months having -0.38 kg (95% CI: -0.64, -0.11) less weight retention [23]. In addition, the effect of breastfeeding had a larger impact on post-partum weight retention if women were less than 30 years old, primipara or had normal pre-pregnancy BMI [23]. These studies evaluated weight status either while mother was breastfeeding or up to one year post breastfeeding; it is interesting that our research has similar findings for weight retention up to 10 years postpartum.

Our results show that breastfeeding for six months is associated with a modest decrease in long-term post-partum weight retention. However, other behavioral drivers may have a greater impact over the longer postpartum period. For example, Lyu et al. [29], found that dietary energy intake could explain 24% of postpartum weight retention variation at 6 months and 27% at 1 year. Over a longer period, excess energy intake could have an even larger impact on weight retention. Another variable impacting weight retention over time is physical activity [30]. Physical activity is often self-reported and difficult to measure but may affect associations between breastfeeding and weight retention; yet it is not always evaluated in weight retention studies [31]. Women who did not exercise had three times the odds of post-partum weight retention at 24 months compared to those who exercised five or more days per week in the past month [26]. Research on long-term weight retention in postpartum women should include assessments of dietary intake and general measures of physical activity to have a more complete picture of factors driving weight retention.

Strengths of the study included the representative nature of the sample and the long-term follow-up. One limitation includes recall bias as breastfeeding duration was asked at the 4–10-year postpartum visit, which some participants may not remember exact dates when they stopped breastfeeding. A second limitation relates to the self-reports of anthropometric measurements, as women tend to underestimate their weight while overestimating their height [32]. As both pre-pregnancy and 4–10 years postpartum weight measurements are self-report, underestimation is likely present in both the pre- and post- pregnancy weights. Due to the nature of this study, body composition measurements beyond weight and length were unable to be assessed. We and others have previously observed that women, even without weight retention, had a 30% increase in visceral adipose tissue from 15 weeks gestation to 59 weeks postpartum [33]; however, breastfeeding was not assessed in this report. Despite our findings of differences of weight retention in the long-term postpartum period, we do not know the body composition distribution and whether breastfeeding impacted central adiposity. There are many ways to calculate SES and our measure may not have captured the full depth of lower socioeconomic status as demonstrated in a recent study looking at different measures of socioeconomic status and poverty [34] including longitudinal measures [35]. No survey questions were asked regarding formula status or age of introduction of foods.

## Conclusion

Six months of breastfeeding was associated with lower body weight and BMI at the 4–10-year postpartum visit. In constrast to our hypothesis, effects did not vary by SES status. Further research is needed to identify the impact of additional behavioral and environmental factors on long-term maternal weight status. Maternal weight status is a predictor of their offspring health well into adulthood. Understanding the drivers for excessive weight retention can help us not only improve the mother’s health but the health of their children .

## Data Availability

The GROW data are not publicly unavailable because of IRB restrictions.
